# Fibroproliferative response to urothelial failure obliterates the ureter lumen in a mouse model of prenatal congenital obstructive nephropathy

**DOI:** 10.1038/srep31137

**Published:** 2016-08-11

**Authors:** Amanda J. Lee, Noemi Polgar, Josephine A. Napoli, Vanessa H. Lui, Kadee-Kalia Tamashiro, Brent A. Fujimoto, Karen S. Thompson, Ben Fogelgren

**Affiliations:** 1 Department of Anatomy, Biochemistry and Physiology, John A. Burns School of Medicine, University of Hawaii at Manoa, HI 96813, USA; 2 Department of Pathology, John A. Burns School of Medicine, University of Hawaii at Manoa, HI 96813, USA

## Abstract

Congenital obstructive nephropathy (CON) is the most prevalent cause of pediatric chronic kidney disease and end-stage renal disease. The ureteropelvic junction (UPJ) region, where the renal pelvis transitions to the ureter, is the most commonly obstructed site in CON. The underlying causes of congenital UPJ obstructions remain poorly understood, especially when they occur *in utero,* in part due to the lack of genetic animal models. We previously showed that conditional inactivation of *Sec10*, a central subunit of the exocyst complex, in the epithelial cells of the ureter and renal collecting system resulted in late gestational bilateral UPJ obstructions with neonatal anuria and death. In this study, we show that without *Sec10*, the urothelial progenitor cells that line the ureter fail to differentiate into superficial cells, which are responsible for producing uroplakin plaques on the luminal surface. These Sec10-knockout urothelial cells undergo cell death by E17.5 and the urothelial barrier becomes leaky to luminal fluid. Also at E17.5, we measured increased expression of TGFβ1 and genes associated with myofibroblast activation, with evidence of stromal remodeling. Our findings support the model that a defective urothelial barrier allows urine to induce a fibrotic wound healing mechanism, which may contribute to human prenatal UPJ obstructions.

Congenital anomalies of the kidney and urinary tract (CAKUT) are a heterogeneous spectrum of disorders that include renal dysplasia, aplasia, ureteral duplication, horseshoe kidney, and obstructions along the urinary tract. Within CAKUT, congenital obstructive nephropathy (CON) is the most common cause of chronic renal failure and end stage renal disease for infants and children^1^,^2^,^3^. The obstructed outflow of urine leads to hydronephrosis, which is the enlargement of the kidney due to a build up of urine in the renal pelvis. Detection of CON often occurs during prenatal development, and hydronephrosis is detected by ultrasound in ~1% of pregnancies^4^,^5^,^6^. Although about 70% of prenatal hydronephrosis cases spontaneously resolve, the remaining 30% of cases can worsen and cause lasting renal damage^6^. The severe cases require surgical correction, such as stent placement or a pyeloplasty. The most common site of obstruction in CON is at the ureteropelvic junction (UPJ), which is where the kidney pelvis transitions into the ureter^7^,^8^,^9^. The underlying molecular causes of congenital UPJ obstructions are poorly understood, as are the contributing environmental factors and natural variability. The gap in knowledge about CON and UPJ obstructions is in part due to the lack of genetic, non-surgical animal models to study these diseases^8^,^10^.

Ureter development in mice begins at embryonic day 10.5 (E10.5) when the ureteric bud grows from the nephric duct in response to signals from the metanephric mesenchyme. As the tip of the ureteric bud grows and branches into the metanephric mesenchyme to become the kidney, the stalk elongates to form the ureter. The base of the ureteric bud migrates down the nephric duct and eventually connects directly with the bladder^11^. Although the ureteric bud starts as a single monolayer of epithelial cells, it induces development of an outer smooth muscle layer and then differentiates into a multilayered transitional epithelium called the urothelium. Lining the renal pelvis, ureter, bladder and urethra, the urothelium is both flexible and fluid impermeable to allow stretching while preventing urine from escaping the lumen of the urinary tract. In order to do this, the lumen-facing superficial cells of the urothelium produce very high levels of transmembrane proteins called uroplakins, which form hexagonal plaques on the luminal surface^12^,^13^,^14^,^15^. These uroplakin plaques cover a majority of the apical plasma membrane and are connected by flexible hinge regions. The four members of the uroplakin family (Upk1a, Upk1b, Upk2, and Upk3) are transmembrane proteins that assemble into heterodimers in the ER (Upk1a-Upk2 and Upk1b-Upk3) before being trafficked through the Golgi and to the apical surface in discoid/fusiform vesicles^13^. A pool of these uroplakin-containing vesicles are maintained under the apical surface of the mature superficial cells, and the dynamic exocytosis and endocytosis allows the urothelial surface area to expand and contract in response to mechanical stretch^16^,^17^. High expression of the uroplakin family members and formation of these plaques is critical for the development and maintenance of the urothelial barrier.

We recently generated a novel transgenic mouse model of prenatal UPJ obstructions by knocking out *Sec10* in ureteric bud-derived epithelial cells, including the kidney distal tubules and collecting duct, renal pelvis, and the ureter^18^. *Sec10* is a central subunit of the octameric exocyst protein complex, which mediates the targeting and docking of intracellular vesicles to some subcellular locales^19^. Previous studies have shown that Sec10 links Sec15, the exocyst subunit that binds specific Rab GTPases on the surface of secretory vesicles, to the rest of the exocyst complex at the plasma membrane^20^,^21^. As previously reported, we crossed the floxed Sec10 mice with the *Ksp-Cre* mouse strain^22^,^23^, where Cre recombinase expression is driven by a 1.3 kb promoter fragment of kidney-specific cadherin (Ksp-cadherin; cadherin 16). During nephrogenesis, Ksp-cadherin is expressed in the ureteric bud and the epithelial cells derived from the ureteric bud, allowing us to investigate the role of Sec10 in these cells during urinary tract development. We showed that 95% of the *Sec10* knockout pups (*Sec10*^*FL/FL*^*;Ksp-Cre*) died hours after birth with severe bilateral hydronephrosis and complete anuria^18^.

These mice had bilateral UPJ obstructions late in gestation, between E17.5 and E18.5, due to a cellular overgrowth that filled the ureter lumen^18^. This overgrowth was composed of mesenchymal shaped cells positive for smooth muscle actin (SMA), with an almost complete disappearance of E-cadherin-positive urothelial cells. Analysis of the *Sec10*^*FL/FL*^*;Ksp-Cre* ureters at E17.5 identified an absence of uroplakin-3 (Upk3) on the superficial surface of the urothelium. In addition, a higher proliferation rate of SMA-positive cells was measured at the UPJ region in these ureters at E17.5, prior to the obstruction of the ureter lumen.

The purpose of this study was to identify the cellular mechanism that causes the *in utero* UPJ obstructions after the conditional inactivation of *Sec10* in epithelial cells of the ureteric bud. Here, we show that the urothelium in *Sec10*^*FL/FL*^*;Ksp-Cre* ureters fails to develop a superficial cell layer and luminal uroplakin plaques between E16.5 and E17.5. In these developing mutant ureters, we measured almost no uroplakin gene expression, and a highly decreased expression of *peroxisome proliferator-activated receptor gamma* (*PPARγ*) at E16.5. PPARγ is a key transcriptional activator of the uroplakin gene family, and has been shown to be critical for urothelial differentiation^24^,^25^,^26^. This suggested that the terminal differentiation of superficial cells is impaired in these mutant embryos. By E17.5, *Sec10* mutant urothelial cells started to undergo cell death and detached from the wall of the ureter, and had largely disappeared by E18.5. Concomitant with the failure of the urothelial barrier by E17.5, we saw increased levels of *TGF-β*1 and other fibrotic markers, invasion of the lumen by fibroblastic cells, as well as rearrangement of the basement membrane with an increased ECM deposition. These results show that in our *Sec10*^*FL/FL*^*;Ksp-Cre* mouse model of prenatal CON, the failure of urothelial differentiation precedes a fibroproliferative wound healing response that occludes the lumen at the UPJ.

## Results

### Prenatal UPJ obstructions in Sec10^FL/FL^;Ksp-Cre mice are preceded by a loss of ureter urothelium

**Figure 1:**
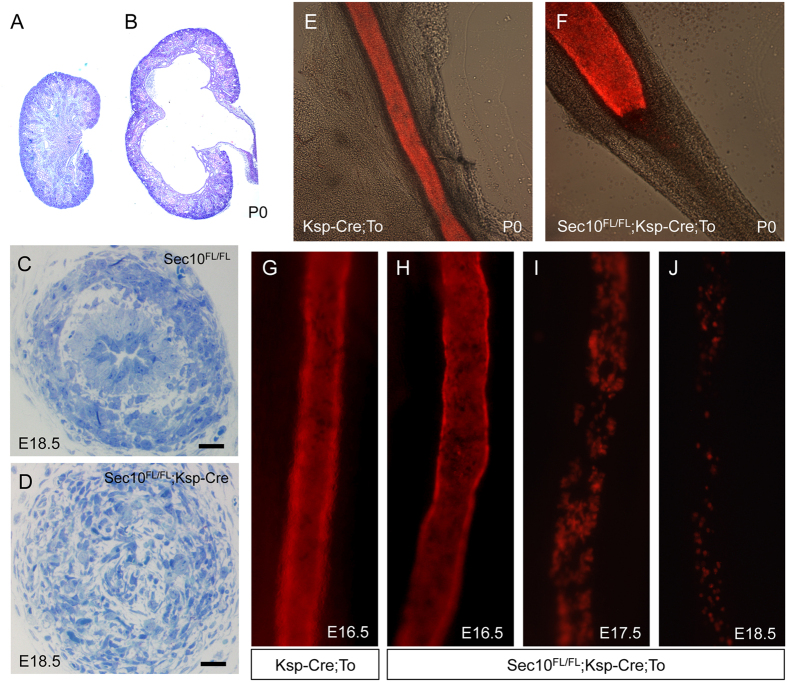
*Sec10*^*FL/FL*^*;Ksp-Cre* ureters form complete UPJ obstructions by E18.5 with loss of urothelial cells starting at E17.5. (**A,B**) Representative H&E-stained histological sections demonstrate substantial hydronephrosis in *Sec10*^*FL/FL*^*;Ksp-Cre* newborn kidneys (**B**), not present in *Sec10*^*FL/FL*^ control littermates (**A**). (**C,D**) Alcian blue staining of cross-sections from representative *Sec10*^*FL/FL*^ and *Sec10*^*FL/FL*^*;Ksp-Cre* ureters at the UPJ region. Bar = 20 μm. (**E,F**) Fluorescence imaging merged with differential interference contrast (DIC) microscopy of representative P0 *Ksp-Cre;To* control and *Sec10*^*FL/FL*^*;Ksp-Cre;To* mutant ureters. Loss of tdTomato-labeled urothelial cells is evident at and below the UPJ obstruction in *Sec10*^*FL/FL*^*;Ksp-Cre;To* ureters. (**G-J**) Fluorescence microscopy of whole mount E16.5 *Ksp-Cre;To* control ureters, and of E16.5-E18.5 *Sec10*^*FL/FL*^*;Ksp-Cre;To* mutant ureters, showing progressive loss of tdTomato-labeled urothelial cells starting after E16.5.

As previously reported, we crossed our novel floxed *Sec10* mouse line with the *Ksp-Cre* mouse strain to conditionally knockout the *Sec10* gene in epithelial cells of the urinary tract derived from the ureteric bud. The *Sec10*^*FL/FL*^*;Ksp-Cre* mice developed bilateral *in utero* UPJ obstructions, severe hydronephrosis (Fig. 1A,B), with neonatal anuria and death, with a 95% penetrance^18^. We observed that the ureter lumen became obstructed at the UPJ region between E17.5 and E18.5, but the underlying basis of the blockage was unclear. By immunostaining for E-cadherin, we saw that epithelial cells had largely disappeared from the obstructed UPJ by E18.5. Representative cross sections of E18.5 ureters stained with Alcian blue show a normal multilayered ureter with a patent lumen in littermate controls (Fig. 1C), but show that *Sec10*^*FL/FL*^*;Ksp-Cre* ureters were completely obstructed by E18.5 (Fig. 1D). From histological analysis, the *Sec10*^*FL/FL*^*;Ksp-Cre* ureters had completely lost the urothelial cell layer by E18.5, with what looked like granulation tissue filling the lumen of the ureters. We utilized a *tdTomato* reporter mouse strain to confirm Cre activity and to track *Sec10* knockout cells in the urothelium. We previously showed that Cre is activated in the Ksp-Cre ureteric bud cells prior to E13.5^18^, confirming an early deletion of the *Sec10* gene during nephrogenesis. As expected, newborn control mice with both *Ksp-Cre* and *tdTomato* alleles exhibited strong red fluorescence in the urothelium of the pelvis and throughout the entire length of the ureter (Fig. 1E). However, in newborn *Sec10*^*FL/FL*^*;Ksp-Cre* mice, red fluorescent cells were visible only in the upper-most ureter (Fig. 1F). As the renal pelvis transitions into the ureter at the UPJ, tdTomato labeling of the urothelial cells revealed an abrupt disappearance of these cells in the *Sec10*^*FL/FL*^*;Ksp-Cre* ureters (Fig. 1F). Whole mount images of younger tdTomato-labeled ureters (E16.5–E18.5) also showed that the number of urothelial cells in *Sec10*^*FL/FL*^*;Ksp-Cre;To* ureters was significantly decreased at E17.5, and by E18.5 there were very few urothelial cells remaining (Fig. 1G–J). This shows that the loss of *Sec10* in urothelial cells leads to degeneration of the urothelial layer prior to the formation of the UPJ obstruction. Also, these data showed that epithelial-mesenchymal transition (EMT) does not contribute to the obstruction in this mouse model, since we did not detect any tdTomato-labeled mesenchymal cells among the tissue filling the ureter lumens.

### Sec10 is necessary for normal differentiation of the superficial urothelial cells in the developing ureter

**Figure 2:**
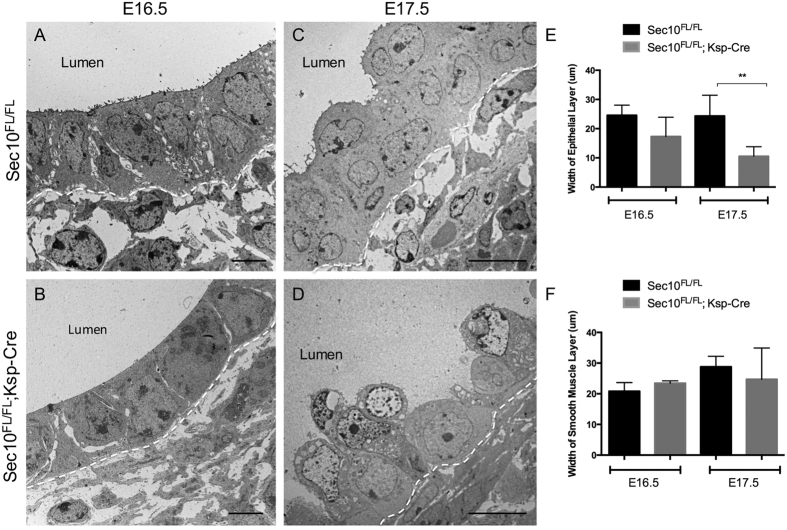
Ultrastructural analysis reveals failure of *Sec10*^*FL/FL*^*;Ksp-Cre* urothelial cells to stratify between E16.5 and E17.5. (**A,B**) Transmission electron microscopy (TEM) of the urothelial layer of E16.5 ureters of *Sec10*^*FL/FL*^*;Ksp-Cre* and *Sec10*^*FL/FL*^ control littermates. Dashed line marks the basement membrane. Bar = 4 μm. (**C,D**) TEM of the urothelial layer of E17.5 ureters of *Sec10*^*FL/FL*^*;Ksp-Cre* and *Sec10*^*FL/FL*^ control littermates. Bar = 10 μm. (**E**) Measurements of urothelial layer thickness in *Sec10*^*FL/FL*^*;Ksp-Cre* versus *Sec10*^*FL/FL*^ ureters at E16.5 and E17.5 (**p < 0.01). (**F**) Measurements of smooth muscle layer thickness in E16.5 and E17.5 ureters based on E-cadherin and smooth muscle actin immunostaining^18^. No significant changes were detected in thicknesses of smooth muscle layers.

Urothelial cell differentiation and stratification during ureter development is critical for the formation of the mature urothelial barrier against urine. The urothelial progenitor epithelial cells, derived from the stalk of the ureteric bud, are initially present as a monolayer, but as they respond to morphogens from surrounding mesenchymal tissue they stratify into three urothelial cell types: basal cells, intermediate cells, and superficial cells^11^,^27^. We used transmission electron microscopy (TEM) to compare the ultrastructure of the ureter’s urothelium in E16.5 and E17.5 *Sec10*^*FL/FL*^ and *Sec10*^*FL/FL*^*;Ksp-Cre* embryos. At E16.5, control ureters had a single urothelial layer with microvilli extending into the lumen of the ureter (Fig. 2A), which looked similar in *Sec10*^*FL/FL*^*;Ksp-Cre* ureters except for a distinct reduction in apical microvilli (Fig. 2B). In both E16.5 *Sec10*^*FL/FL*^*;Ksp-Cre* and control ureters, projections were visible between the urothelial cells, an early sign of epithelial stratification. At E17.5, control ureter cross sections revealed a two-layered urothelium with a characteristic scalloped structure with hinge regions on the luminal membrane of the superficial cells (Fig. 2C). However, E17.5 *Sec10*^*FL/FL*^*;Ksp-Cre* ureters showed a highly abnormal single urothelial layer, with gaps in the epithelium and cells pulling away from the basement membrane toward the center of the lumen (Fig. 2D). These Sec10-knockout urothelial cells had lost large amounts of cytoplasm, and showed irregular disrupted plasma membranes and unusual distributions of electron-dense material in the nuclei. From immunostaining E-cadherin and SMA in E16.5 and E17.5 ureter cross sections, we measured the widths of both urothelial and smooth muscle layers in *Sec10*^*FL/FL*^*;Ksp-Cre* and *Sec10*^*FL/FL*^ ureters. We confirmed that at E16.5, *Sec10*^*FL/FL*^*;Ksp-Cre* ureters had no significant difference in the average width of the urothelial layer compared to *Sec10*^*FL/FL*^ ureters, but by E17.5, the Sec10-knockout urothelial layer was about half the width of control urothelium (Fig. 2E). No significant changes in the width of the smooth muscle layer at either E16.5 or E17.5 were measured (Fig. 2F).

**Figure 3:**
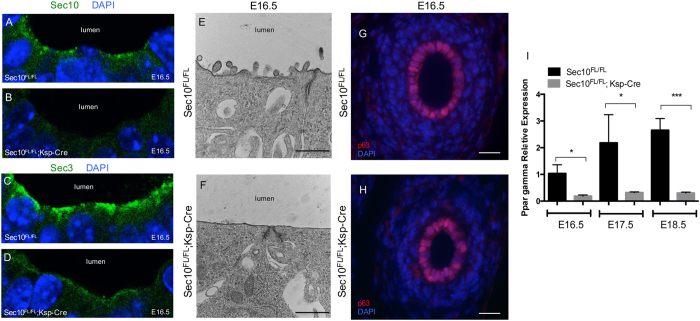
Loss of *Sec10* in urothelial cells results in defective urothelial cell differentiation and stratification. (**A–D**) Immunostaining and confocal microscopy of exocyst members Sec10 and Sec3 in E16.5 ureters revealed exocyst was localized at the apical/luminal plasma membrane. The Sec10 protein was absent in E16.5 *Sec10*^*FL/FL*^*;Ksp-Cre* urothelial cells compared to *Sec10*^*FL/FL*^ ureters (**A,B**), and Sec3 was also significantly decreased in *Sec10*^*FL/FL*^*;Ksp-Cre* ureters. (**E,F**) TEM of the apical plasma membrane of E16.5 *Sec10*^*FL/FL*^*;Ksp-Cre* and *Sec10*^*FL/FL*^ urothelial cells at 6000x. Bar = 500nm. (**G,H**) Immunohistochemistry of p63 (red) in E16.5 *Sec10*^*FL/FL*^*;Ksp-Cre* and *Sec10*^*FL/FL*^ ureters showed no detectable differences. (**I**) Real time qPCR measurement of *PPARγ* gene expression in E16.5–E18.5 *Sec10*^*FL/FL*^*;Ksp-Cre* and *Sec10*^*FL/FL*^ ureters (*p < 0.05; ***p < 0.001). C_t_ values for each gene were normalized against *beta actin*.

Confocal imaging of E16.5 ureter cross sections after immunohistochemistry revealed that members of the exocyst complex, Sec10 and Sec3, localize at the apical (luminal) plasma membrane in *Sec10*^*FL/FL*^ urothelial cells (Fig. 3A,C). As expected, in *Sec10*^*FL/FL*^*;Ksp-Cre* mutant ureters, Sec10 was completely absent, but also Sec3 levels were significantly decreased (Fig. 3B,D). Degradation of other exocyst subunits was previously measured in Sec10-knockdown MDCK cells^28^, but this is the first *in vivo* evidence that the Sec10 protein may be required for the expression or stability of the other exocyst subunits. These data also showed that the localization of the exocyst in these monolayered urothelial cells at E16.5 differs from previous reports of the exocyst in other monolayered epithelial cells, where it localized to sites of cell-cell contact and had been associated with basolateral membrane delivery^21^,^29^,^30^. The decrease in exocyst protein at the apical plasma membrane in E16.5 *Sec10*^*FL/FL*^*;Ksp-Cre* urothelial cells coincided with highly decreased number of microvilli on the apical surface (Fig. 3E,F). The transcription factor *p63* is highly expressed in the progenitor ureteric bud and has been shown to be important in the growth and differentiation of epithelial tissues^31^,^32^. In the mature urothelium, it is highly expressed in the basal urothelial cells and is critical for the maintenance the basal layer^26^,^33^. Immunohistochemistry of *Sec10*^*FL/FL*^ and *Sec10*^*FL/FL*^*;Ksp-Cre* ureters showed similar *p63* levels and localization at E16.5 (Fig. 3G,H). PPARγ is a nuclear receptor that is a critical activator of uroplakin gene expression and has been reported to be necessary for the differentiation of superficial urothelial cells^24^,^25^,^26^. With qPCR, we measured a large decrease in *PPARγ* gene expression in *Sec10*^*FL/FL*^*;Ksp-Cre* ureters compared to *Sec10*^*FL/FL*^ ureters from E16.5 to E18.5 (Fig. 3I). That *PPARγ* was 80% decreased in *Sec10*^*FL/FL*^*;Ksp-Cre* ureters as early at E16.5, prior to gross morphological changes, supports the hypothesis that *Sec10* is required for the differentiation of superficial urothelial cells in embryonic ureters.

### Sec10-knockout urothelial cells fail to produce uroplakin plaques on the luminal surface

**Figure 4:**
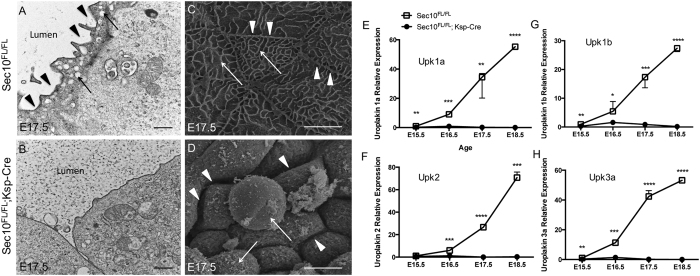
Electron microscopy and real time qPCR of E17.5 *Sec10*^*FL/FL*^*;Ksp-Cre* ureters confirms an absence of uroplakin plaques on the luminal surface of urothelial cells. (**A,B**) TEM of E17.5 *Sec10*^*FL/FL*^ control ureters detected scallop-shaped uroplakin plaques on the apical plasma membrane (arrow heads) and fusiform vesicles being trafficked to the apical surface (arrows), both characteristic of superficial cells. In *Sec10*^*FL/FL*^*;Ksp-Cre* ureters, no uroplakin plaques or vesicles were detected. Bar = 0.5 μm. (**C,D**) Scanning electron microscopy (SEM) of the luminal surfaces of E17.5 *Sec10*^*FL/FL*^ control ureters showed hallmark hexagonal uroplakin plaques covered the surface (indicated by arrows), with well-established tight cell-cell junctions (indicated by arrow heads). SEM of the luminal surface of E17.5 *Sec10*^*FL/FL*^*;Ksp-Cre* ureters showed a complete absence of uroplakin plaques and damaged cells pulling away from the urothelial layer (indicated by arrows) along with poor cell-cell junctions (indicated by arrow heads). Bar = 5 μm. (**E–H**) Real time qPCR measurement of *Upk1a*, *Upk1b*, *Upk2*, and *Upk3a* gene expression in *Sec10*^*FL/FL*^*;Ksp-Cre* and *Sec10*^*FL/FL*^ ureters collected from E15.5–E18.5 embryos (*p < 0.05, **p < 0.01, ***p < 0.001, ****p < 0.0001). C_t_ values for each gene were normalized against *beta actin*.

Differentiation of superficial urothelial cells has been shown, in the bladder, to be an early event in urothelial maturation^33^. Normal mature superficial cells are characterized by the presence of numerous intracellular vesicles carrying uroplakins at the luminal plasma membrane, which establishes and maintains the watertight barrier against urine. Previously, we detected an absence of Upk3 protein in the *Sec10*^*FL/FL*^*;Ksp-Cre* urothelium at E17.5 via immunohistochemistry^18^. Here we investigated production of uroplakins in *Sec10*^*FL/FL*^*;Ksp-Cre* urothelium in more detail. TEM of control ureters at E17.5 showed numerous intracellular vesicles that clustered towards the apical membrane of the superficial urothelial cells (arrows, Fig. 4A). However, in the E17.5 *Sec10*^*FL/FL*^*;Ksp-Cre* urothelial cells, there was a complete absence of these apical vesicles (Fig. 4B), correlating with the absent uroplakin mRNA gene expression that we measured by qPCR (Fig. 4E–H). Using scanning electron microscopy (SEM) of E17.5 ureter lumens, we could clearly visualize the hexagonal uroplakin plaques on the luminal membrane surface of *Sec10*^*FL/FL*^ control ureters and tight cell-cell contacts (Fig. 4C). SEM also confirmed that uroplakin plaques were completely missing on the luminal surface of E17.5 *Sec10*^*FL/FL*^*;Ksp-Cre* ureters (Fig. 4D). We also noted that the *Sec10*^*FL/FL*^*;Ksp-Cre* urothelial cells did not show tight cell-cell contacts at E17.5, which confirms findings with TEM that many of these cells are damaged and are becoming detached from the ureter wall.

### Necrosis of urothelial cells contributes to the loss of barrier integrity in Sec10^FL/FL^;Ksp-Cre ureters

**Figure 5:**
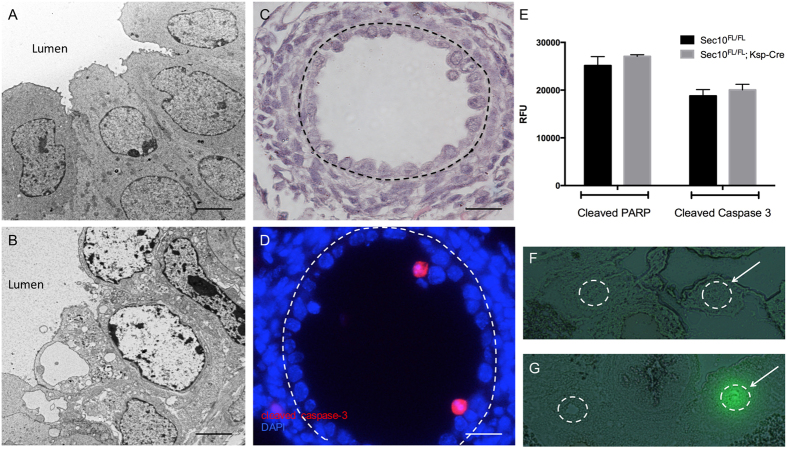
Loss of urothelial cells at E17.5 is primarily due to necrosis and not apoptosis. (**A,B**) Representative TEM of the urothelial layer of E17.5 *Sec10*^*FL/FL*^ and *Sec10*^*FL/FL*^*;Ksp-Cre* ureters. Most of the remaining attached urothelial cells in *Sec10*^*FL/FL*^*;Ksp-Cre* ureters displayed characteristics of cell damage and necrosis, but not apoptosis. Bar = 4 μm. (**C,D**) Serial sections of a representative E17.5 *Sec10*^*FL/FL*^*;Ksp-Cre* ureter (dotted line depicts the basement membrane) demonstrated that although the urothelium was a damaged single layer (H&E staining, **C**), very few cells were positive for activated caspase-3 (red, **D**). Bar = 20 μm. (**E**) Measurement of cleaved caspase-3 and cleaved PARP levels in multiple *Sec10*^*FL/FL*^ and *Sec10*^*FL/FL*^*;Ksp-Cre* ureters did not detect any significant increase in these apoptotic markers at E17.5. Columns represent the means of relative fluorescent units (RFU), with error bars representing SEM. (**F,G**) In E17.5 embryos, the left renal pelvis was injected with FITC-dextran which was allowed to flow to the bladder, then tissue was frozen and cryosectioned (ureters are circled, arrows note left ureter). Normal *Sec10*^*FL/FL*^ controls (**F**) had very little FITC-dextran retained in the injected ureter (arrow), but *Sec10*^*FL/FL*^*;Ksp-Cre* mice (**G**) had a much larger retention of the dextran in the injected ureter tissue, indicating leakiness of the urothelial barrier.

Analysis of E17.5 *Sec10*^*FL/FL*^*;Ksp-Cre* TEM images revealed that the damaged cells pulling away from the basement membrane had morphological characteristics similar to necrosis rather than apoptosis (Fig. 5A,B). Apoptotic cells via TEM exhibit membrane blebbing and nuclear fragmentation, but necrotic cells showed a darkening of the nucleus, uncontrolled swelling of the cell, rough irregular plasma membranes, and an absence of blebbing. Immunohistochemistry of E17.5 ureter cross-sections confirmed that very few urothelial cells were positive for cleaved caspase 3 (Fig. 5C,D), a marker for apoptotic activation. Protein analysis of *Sec10*^*FL/FL*^*;Ksp-Cre* and *Sec10*^*FL/FL*^ ureters also confirmed no significant differences between the levels of cleaved caspase-3 or cleaved PARP, another marker of apoptosis (Fig. 5E). The absence of apoptotic characteristics, and the cell morphology observed via TEM, indicated that *Sec10-knockout* urothelial cells in the developing ureter were dying largely due to necrosis.

Based on TEM images of the disrupted urothelium at E17.5, we performed fluorescein isothiocyanate dextran (FITC-dextran) injections into the renal pelvis of the kidney to determine if there was any sign of urothelial barrier dysfunction in the ureter. In control E17.5 ureters (injected ureter marked by arrow, Fig. 5F), the FITC-dextran was not retained in the ureters and passed through the ureters and into the bladders, indicating a strong luminal barrier. In contrast, E17.5 *Sec10*^*FL/FL*^*;Ksp-Cre* ureters retained a very strong green fluorescence in the injected ureters (arrow, Fig. 5G), indicating the retention of the FITC-dextran in the tissue. Thus, the disrupted differentiation of the Sec10-knockout urothelium has a functional consequence of compromising the urothelial barrier by E17.5.

### Urothelial degeneration in the developing Sec10^FL/FL^;Ksp-Cre ureter induces a fibroproliferative response that rapidly occludes the lumen

**Figure 6:**
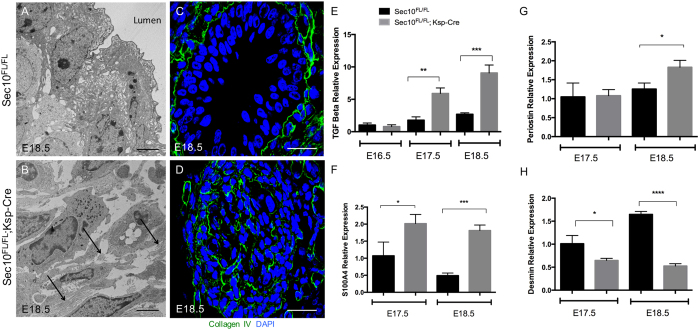
Obstructed *Sec10*^*FL/FL*^*;Ksp-Cre* ureters have stromal remodeling and increased expression of *TGFβ1* and other fibroblastic markers. (**A,B**) TEM of ureter cross sections at E18.5. *Sec10*^*FL/FL*^ sections show mature superficial urothelial cells with uroplakin plaques on the apical membrane. In *Sec10*^*FL/FL*^*;Ksp-Cre* ureters, the lumen has been filled with fibroblastic cells and extracellular matrix deposits. Arrows mark collagen fibers in the filled lumen. Bar = 4 μm. (**C**) Immunohistochemistry of collagen IV in a *Sec10*^*FL/FL*^ E18.5 ureter cross section revealed a distinct basement membrane separating the epithelial and smooth muscle layers. (**D**) Collagen IV immunohistochemistry in *Sec10*^*FL/FL*^*;Ksp-Cre* E18.5 ureter cross sections revealed a remodeled basement membrane and no distinction between cell layers. Bar = 20 μm. (**E–H**) Real time qPCR analysis of *TGFβ1, S100A4* (*fibroblast specific protein*), *periostin*, and *desmin* relative gene expression in *Sec10*^*FL/FL*^*;Ksp-Cre* and *Sec10*^*FL/FL*^ embryonic ureters. *p < 0.05, **p < 0.01, ***p < 0.001, ****p < 0.0001. C_t_ values for each gene were normalized against *beta actin*.

In histological sections of our *Sec10* knockout ureter cross sections, we noted that the tissue in the UPJ obstruction looked similar to granulation tissue, characteristic of wound healing. Granulation tissue includes the presence of myofibroblasts, extracellular matrix (ECM) deposition and remodeling, inflammatory cells, and newly formed capillaries. Although often associated with wound healing of the skin, the fibroproliferative response seen in granulation tissue can also occur in internal epithelial tissues to obliterate lumens, such as in bronchiolitis obliterans^34^. Here, we expanded on our original findings and performed ultrastructure morphological and molecular analysis at the UPJ obstruction to evaluate the degree of fibrotic response. TEM analysis of control *Sec10*^*FL/FL*^ ureters at E18.5 confirmed a mature multilayered structure with numerous uroplakin vesicles (Fig. 6A), and a distinct basement membrane (data not shown). However, *Sec10*^*FL/FL*^*;Ksp-Cre* ureters exhibited a complete loss of the epithelial layer, previously shown by immunostaining for epithelial markers such as E-cadherin^18^. Instead, the lumen was filled with migrating fibroblastic cells and deposits of ECM (Fig. 6B), suggesting the presence of a fibrotic wound healing response at the obstruction. Immunohistochemistry of collagen IV in *Sec10*^*FL/FL*^ ureters showed a distinct basement membrane attached to the basal layer of the urothelium separating the mucosa from the smooth muscle cells (Fig. 6C). In contrast, *Sec10*^*FL/FL*^*;Ksp-Cre* ureters had a reorganization of collagen IV throughout the new tissue in the lumen of the ureter, indicating a disrupted and expanded basement membrane that allowed the surrounding mesenchyme to penetrate the lumen (Fig. 6D).

*TGF-β1* is a critical mediator of wound healing and fibrosis, and was found in a previous study to be increased in human UPJ samples^35^. We hypothesized that the TGFβ pathway may be activated from urinary leakage into the tissue underlying the disrupted urothelium in *Sec10*^*FL/FL*^*;Ksp-Cre* ureters. We measured *TGF-β1* mRNA expression using real time qPCR in E16.5-E18.5 *Sec10*^*FL/FL*^*;Ksp-Cre* ureters and compared to *Sec10*^*FL/FL*^controls. *TGF-β1* had a five-fold expression increase in *Sec10*^*FL/FL*^*;Ksp-Cre* at E17.5 and E18.5 compared to *Sec10*^*FL/FL*^ureters (Fig. 6E). At E16.5 however, prior to the observed urothelial degeneration, we measured no change in *TGF-β1* expression. We had previously reported an over proliferation of cells positive for SMA in the surrounding mesenchyme at E17.5 prior to the onset of the obstruction^18^. However, multiple cell types express SMA, including smooth muscle cells and activated myofibroblasts. The presence of activated fibroblasts (myofibroblasts) is one of the key characteristics of the wound healing response. Quantitative PCR analysis showed a significant increase in expression of S100A4 (*fibroblast specific protein*, FSP) at E17.5 and E18.5 (Fig. 6F). There was also a significantly increased level of *periostin* expression at E18.5 (Fig. 6G), but decreased *desmin* expression in the ureters at E17.5 and E18.5 (Fig. 6H). Periostin is a matricellular protein that has been shown to promote myofibroblast proliferation and differentiation during wound healing and fibrosis^37^,^38^,^39^. In contrast, desmin is specific to smooth muscle cells and is not expressed in myofibroblasts. This expression data indicated that the cells invading the ureter lumen in *Sec10*^*FL/FL*^*;Ksp-Cre* embryos were activated myofibroblasts, rather than an expanded smooth muscle cell population. In summary, the evidence supports our hypothesis that the UPJ obstruction arises in these *Sec10* mutant mice from a fibroproliferative wound healing response after degeneration of the urothelium in the embryonic ureter.

## Discussion

In this study, we identify the underlying cellular basis of the *in utero* UPJ obstruction, and a timeline of ureter maldevelopment, in a *Sec10* conditional knockout mouse. We provide evidence that Sec10 is necessary for proper differentiation of superficial urothelial cells and establishment of the uroplakin barrier. It is not clear what exactly causes the cell death observed in E17.5 *Sec10*^*FL/FL*^*;Ksp-Cre* urothelium, but our data shows that it is primarily necrotic in origin. Since the *Sec10* deletion occurs in these cells prior to E13.5^18^, we hypothesize that the cell death after E16.5 is either in response to a failure of superficial differentiation or the lack of protection against the growing urine flow, or likely a combination of multiple factors. We also hypothesize that the leakage of urine through the urothelial barrier induces the observed fibrotic wound healing response from the surrounding mesenchyme. We measured no change in *TGF-β1* expression at E16.5 in *Sec10*^*FL/FL*^*;Ksp-Cre* ureters, prior to the urothelial cell death and barrier degeneration. But beginning at E17.5, we measured an increase in *TGF-β1* expression (Fig. 6E), as well as an increase in the fibroblast specific gene *S100A4* (Fig. 6F). We also measured increased expression of *periostin* at E18.5, which is now recognized to be a critical regulator of the activation of fibroblast during wound healing and fibrosis^36^,^37^,^38^. This correlates with a decrease in the relative expression of *desmin*, by the smooth muscle cells. Although the surrounding mesenchyme includes both fibroblasts and smooth muscle cells, our data suggest the resident fibroblast population becomes activated myofibroblasts that rapidly proliferate and migrate to obstruct the ureter lumen between E17.5 and E18.5.

The progression of kidney disease arising from CON has been studied for decades, but despite this, we still know very little about the genetic and cellular basis of human UPJ obstructions. The unilateral ureter obstruction (UUO) animal model, which requires surgery to ligate one ureter, has been the most widely studied model of CON^39^. This model has greatly advanced the current understanding of the stages of renal pathology after the ureter obstruction and after correction of the obstruction. The obstructed kidney develops parenchymal loss and interstitial fibrosis that leads to loss of renal function^40^, a pathology common to many renal diseases. However, surgical models like the UUO are labor intensive with some degree of technical variability, and also limited in that they cannot be used to investigate the natural causes of human UPJ obstructions. Additionally, with the exception of large animals models like sheep^41^, they cannot be used to study ureter obstructions that occur *in utero*.

Few genetic models of CON have been identified and characterized. One of these models is the megabladder mouse, in which the mice develop hydronephrosis and lower urinary tract obstructions secondary to a non-functional over-distended bladder^42^. Other mouse models of CON have arisen from targeted deletion of genes necessary for growth or differentiation of ureter smooth muscle cells or pacemaker cells^43^,^44^. However, these models are non-obstructive, with a failure of ureter muscle tension that leads to hydroureter and hydronephrosis. The models that display UPJ obstructions typically involve inducing over proliferation of the surrounding smooth muscle, but without urothelial degeneration or fibrotic infiltration of the lumen^45^,^46^. None of these mouse models of CON are neonatal lethal and they typically have wide-ranging variability and penetrance. In comparison, this *Sec10*^*FL/FL*^*;Ksp-Cre* model is the most consistently severe, with ~95% of the embryos developing lumen-obliterating bilateral UPJ obstructions with neonatal anuria and death^18^.

Clinical cases of UPJ obstructions, often detected as hydronephrosis via prenatal ultrasound, are highly variable in severity and progression^4^,^5^,^6^. In addition, the lack of prognostic indicators or biomarkers requires ongoing surveillance, which is a burden to the patient and costly^47^,^48^,^49^. Typically, cases of UPJ obstructions can be classified as intrinsic or extrinsic, where the more common intrinsic factors involve malformation of the ureter or cellular overgrowth in the ureter lumen, while extrinsic causes include pressure on the ureter from other tissues such as crossing vessels. One histological study demonstrated that surgically removed human samples of intrinsic obstructed UPJ regions had excessive collagen fibers that replaced smooth muscle in the ureter^50^. Another study of congenital UPJ obstructions from 25 patients (versus 15 age-matched control samples) showed an increase in *TGF-β1* mRNA expression and protein levels in the stenotic segments^35^. Taken together, these studies show that at least for a percentage of human UPJ obstructions, there is a significant increase in fibrosis at the stenotic region. Thus, the molecular and histological findings in our *Sec10* mouse model of UPJ obstructions are consistent with the ureter pathology of a significant portion of human clinical cases of UPJ obstructions.

Based on crosses with our *tdTomato* reporter mice, we know Cre-mediated inactivation occurs prior to E13.5 in *Sec10*^*FL/FL*^*;Ksp-Cre* embryos^18^. However, the first abnormalities that we have detected did not occur until ~E16.5, which demonstrates that Sec10 knockout in these epithelial cells does not *de facto* cause cell death. At E16.5, there was a significant decrease in *PPARγ* expression (Fig. 3I), which is normally expressed at high levels during urothelial differentiation of the superficial cells and regulates transcription of the uroplakin gene family through FOXA1 and IRF-1 mediators^24^,^25^. A *PPARγ* conditional knockout mouse, created using the *Hoxb7-Cre* strain that targets the same ureteric bud derived epithelial cells as the *Ksp-Cre* mice, showed that although *PPARγ* is specifically important for differentiation of superficial urothelial cells, but knockout of *PPARγ* did not cause urothelial cell death or prenatal UPJ obstructions. Both *Upk2* and *Upk3* knockout mice have also been reported, and they had severe defects in uroplakin plaque formation at the apical surface and did not have proper urothelial barrier function^51^,^52^. However, these knockout mice also did not display the *in utero* urothelial degeneration and fibroproliferative UPJ obstructions seen in our *Sec10* knockout model. This suggests that failure of superficial differentiation, or failure to produce uroplakin plaques, is not sufficient to produce our UPJ obstruction phenotype. Thus, the exocyst likely has additional roles in urothelial cells or their progenitors making this *Sec10* knockout model a unique tool to bridge the gap between urothelial differentiation and the onset of UPJ obstructions.

Significant decrease in mRNA expression of the four members of the uroplakin gene family in the *Sec10*^*FL/FL*^*;Ksp-Cre* ureters starting at E16.5 (Fig. 4) indicate that the exocyst regulates superficial cell differentiation. It is also possible that the exocyst plays a direct role in uroplakin vesicle trafficking to the apical membrane. The decreased microvilli on the apical surface of E16.5 *Sec10*^*FL/FL*^*;Ksp-Cre* urothelial cells (Fig. 3E,F) indicates disrupted membrane trafficking toward the apical plasma membrane where *Sec10* and *Sec3* are localized in control ureters (Fig. 3A,C). Although some evidence has suggested the exocyst is primarily a basolateral vesicle regulator in polarized epithelial cells^21^,^29^,^30^, other studies have shown apical trafficking in some epithelial cell types can utilize the exocyst^53^,^54^,^55^. Several previous studies have also firmly established that the exocyst subunit *Sec15* interacts directly with *Rab11* and *Rab8* GTPases to promote exocytosis of vesicles bound to those proteins^53^,^56^,^57^,^58^. *Rab8* and *Rab11* are both present on uroplakin-containing discoidal/fusiform vesicles in bladder superficial cells, and both have been shown to be critical for the stretch-induced exocytosis of new uroplakin plaques^59^,^60^. This established connection between two Rab GTPases, which directly regulate uroplakin exocytosis and recycling, and the exocyst complex suggests that the exocyst also has a direct role in uroplakin exocytosis and dynamic recycling. Utilizing inducible Cre mouse strains that avoid urothelial differentiation defects could allow investigations of uroplakin trafficking in mature Sec10-knockout superficial cells.

Our histological analysis of the obstructed ureter at the UPJ region identified pathological changes similar to granulation tissue, with a robust and rapid fibroproliferative response between E17.5 and E18.5. This included invasion of the lumen by cells with a myofibroblastic appearance, deposition of ECM, and disruption of the basement membrane underlying the epithelium. Based on our *tdTomato* labeling of the Sec10-knockout urothelial cells, and the absence of any red-labeled fibroblastic cells in the obstructed lumens, we ruled out EMT as a contributor to the fibrotic response. We hypothesize the pathogenesis in our model of *in utero* UPJ obstructions shares similarities with other diseases of epithelial injury and aberrant stromal remodeling of internal tissues, such as obliterative bronchiolitis. In this pulmonary pathology commonly associated with lung transplants, the epithelial cell layer lining the bronchioles is damaged, triggering a fibroproliferative response of the underlying stroma that obstructs the airway lumen^34^. Our *Sec10*^*FL/FL*^*;Ksp-Cre* mouse model shows that this mechanism may also contribute to prenatal UPJ obstructions, which may be triggered by leakage of the urine into the ureter’s interstitial tissue. This mechanism for congenital UPJ obstructions was previously hypothesized based on histological observations of human pyeloplasty samples. Although Bartoli *et al.* first published this hypothesis 20 years ago^61^, our model provides the first experimental evidence that defective urothelial maturation during prenatal development can induce a fibroproliferative response from the underlying mesenchyme.

Future investigations will aim to identify exactly how the exocyst intersects with specific genetic signaling pathways and morphogens known to regulate urothelial differentiation and ureter development. Key to our understanding of the role of the exocyst in these processes will be identifying which proteins are trafficked by the exocyst complex in urothelial cells. We will likely find the exocyst is multifunctional in this cell type, as this complex has already been implicated in extremely diverse cellular processes, depending on the cell type and environment. Additionally, it will be important to investigate how the urine may damage the tissue underlying the leaky *Sec10*^*FL/FL*^*;Ksp-Cre* urothelium at E17.5, and test how this damage induces the fibroproliferative response that rapidly occludes the lumen. This has direct implications to clinical cases of both intrinsic and extrinsic UPJ obstructions, and we may be able to use the *Sec10*^*FL/FL*^*;Ksp-Cre* mouse model to screen potential therapeutics that ameliorate this type of response, or identify potential diagnostic or predictive biomarkers. Collectively, findings presented in this study demonstrate this *Sec10*^*FL/FL*^*;Ksp-Cre* mouse model may be highly valuable for extending our understanding of the etiology of human congenital UPJ obstructions and the associated renal disease, as well as identifying novel approaches for treatments.

## Materials and Methods

### Animals

All animal procedures and protocols were carried out in accordance with IACUC specifications approved by the University of Hawaii Animal and Veterinary Services. Dr. Fogelgren’s IACUC approved protocol is #11**-**1094, and the University of Hawaii has an Animal Welfare Assurance on file with the Office of Laboratory Animal Welfare (OLAW), assurance number is A3423-01. Adult mice were housed under standard conditions with 12-hr light cycle and supplied with water and food *ad libitum*. The floxed *Sec10* mouse strain (*Sec10*^*FL/FL*^) was generated and used as previously described^18^. The *Ksp-Cre* mouse strain was obtained from Jackson Laboratories^22^,^23^. The *B6.Cg-Gt(ROSA)26Sor*^*tm9(CAG-tdTomato)Hze*^*/J* reporter mouse strain (here designated *tdTomato* or *To*) was kindly provided by Dr. Michelle Tallquist at University of Hawaii and was used to detect Cre recombinase activity through Cre-activated expression of the tdTomato red fluorescent protein^62^. All mice were of C57Bl/6J inbred background. For timed matings, females mated with a male overnight were examined for a vaginal plug the following morning and constituted gestational day E0.5 if present. Both male and female embryos were obtained between days E13.5 and E18.5 and were subsequently staged using Theiler staging criteria (TS) to ensure the developmental stage of each embryo was similar to the conception day (E) designation^63^. Only animals of the same E designation and TS were compared.

### Electron Microscopy

Dissected kidneys and ureters were fixed with 2.5% glutaraldehyde and in 0.1 M sodium cacodylate buffer, pH 7.2, washed in 0.1 M cacodylate buffer for 3 × 15 min, followed by post fixation with 1% OsO_4_ in 0.1 M cacodylate buffer for 1 hour. The tissue was dehydrated in a graded ethanol series (30%, 50%, 70%, 85%, 95%, 100%), substituted with propylene oxide, and either embedded in LX112 epoxy resin for transmission electron microscopy (TEM) or dried in a Tousimis Samdri-795 critical point dryer for scanning electron microscopy (SEM). For TEM, ultrathin sections (60–80 nm) were obtained on a Reichert Ultracut E ultramicrotome, double stained with uranyl acetate and lead citrate, viewed on a Hitachi HT7700 TEM at 100 kV, and photographed with an AMT XR-41B 2k × 2k CCD camera. For SEM, tissues were mounted on aluminum stubs with double stick tape and ureters were opened using a razor blade, and subsequently coated with gold/palladium in a Hummer 6.2 sputter coater. Tissues were viewed and digital images were obtained with a Hitachi S-4800 Field Emission Scanning Electron Microscope at an accelerating voltage of 5 kV.

### Quantitative real time PCR analysis

Gene expression in embryonic tissues using real time quantitative PCR (qPCR) was performed as described previously, using the 2^(−ΔΔCt) method of analysis to calculate fold changes of expression^64^. Briefly, ureter segments from the UPJ region from embryos of various stages were dissected and placed immediately into RNAlater (Sigma) and stored at −20 °C. Ureters from three animals of the same stage and with the same genotype were pooled and extracted using the RNeasy micro kit (Qiagen). cDNA was generated from extracted RNA using the iSCRIPT reverse transcriptase (Bio-Rad) and qPCR was performed using SYBR green (Bio-Rad) with a CFX96 Real Time System (Bio-Rad), as per the manufacturer’s recommended instructions. Please refer to Supplementary Information for a complete list of primer sequences used for qPCR. Expression data was analyzed using Graphpad Prism software. Differences between means for any parameter measured in two groups of age-matched mice were evaluated using Student’s t-tests.

### Histology and Immunohistochemistry

Caudal torsos of Sec10 knockout and control animals were dissected, the abdominal cavity opened, immediately placed in freshly prepared 4% formaldehyde in PBS, and fixed overnight with rocking at 4 °C. Some samples were embedded in paraffin according to standard methods, and some samples were instead subjected to cryosectioning. The tissues were sectioned into 5 μm sections and staining and immunohistochemistry procedures were performed as previously reported^18^. Primary antibodies used for immunostaining were: anti-cleaved caspase-3 at 1:400 (Cat # 9664, Cell Signaling Tech.); anti-p63 at manufacturer’s prepared dilution (Cat # API 3050 G3, Biocare Medical); anti-collagen IV at 1:200 (Cat # ab6586, Abcam); anti-E-cadherin at 1:200 (Cat # 3195, Cell Signaling Tech.); anti-smooth muscle actin at 1:800 (Cat # A2547, Sigma). Stained sections were analyzed using a fluorescent Olympus BX41 microscope or an Olympus Fluoview1000 confocal microscope. Image processing, quantification of cell layer widths, and cell counts were done using Image J software (NIH). For ureter injections, fluorescein isothiocyanate (FITC) labeled dextran (average molecular weight 10,000, Cat # FD10S, Sigma) was dissolved in PBS to make a stock solution of 25 mg/ml. The FITC-dextran solution was injected into the renal pelvis (left kidney only) of genitourinary tracts and allowed to flow to the bladder. Injected tissues were frozen in OCT, cryosectioned, dried, and analyzed with a fluorescent Olympus BX41 microscope.

### Protein Analysis

Ureters were dissected from E17.5 and E18.5 embryos and immediately frozen. Proteins were extracted using standard RIPA lysis buffer with phosphatase and protease inhibitors. Protein quantification was performed using the Bradford Assay and 1.0 μg/μl of protein lysate was placed onto each chamber of the PathScan Intracellular signaling array (Cell Signaling Technology #7744). Assay conditions and procedures followed the manufacturer’s recommendations. Florescence readout was performed using the Licor Odyssey Imager. PathScan data analysis was quantified using ImageStudio software provided by Licor Biosciences. The relative fluorescence unit (RFU) of each antibody spot was quantified according to the Cell Signaling protocol and normalized to the positive control antibody spots (also provided on the Cell Signaling array). Student t-test comparisons were performed using Prism GraphPad software.

## Additional Information

**How to cite this article**: Lee, A. J. *et al.* Fibroproliferative response to urothelial failure obliterates the ureter lumen in a mouse model of prenatal congenital obstructive nephropathy. *Sci. Rep.* **6**, 31137; doi: 10.1038/srep31137 (2016).

## Supplementary Material

Supplementary Information
