# Supplementary material for: Fibroproliferative response to urothelial failure obliterates the ureter lumen in a mouse model of prenatal congenital obstructive nephropathy
Source: Sci Rep. 2016 Aug 11;6:31137. doi: 10.1038/srep31137 (PMC4980620; doi:10.1038/srep31137)
Supplement: Supplementary Information [file srep31137-s1.pdf]

Fibroproliferative response to urothelial failure obliterates the ureter lumen in a mouse model of prenatal congenital obstructive nephropathy

Amanda J. Lee[1], Noemi Polgar[1], Josephine A. Napoli[1], Vanessa H. Lui[1], Kadee-Kalia Tamashiro[1], Brent A. Fujimoto[1], Karen S. Thompson[2], and Ben Fogelgren[1]*

Affiliations:
[1]*Department of Anatomy, Biochemistry and Physiology, John A. Burns School of Medicine, University of Hawaii at Manoa, HI 96813, USA*
[2]*Department of Pathology, John A. Burns School of Medicine, University of Hawaii at Manoa, HI 96813, USA*

SUPPLEMENTARY INFORMATION

Primer sequences for qPCR analysis:

**Gene**	**Forward Primer (5'-3')**	**Reverse Primer (5'-3')**
Upk1a	AGGCAAGGATGATGTCTTCG	ATGAGCATCAGCAGCAGGTA
Upk1b	CTTGGATAGGCATGTTCGTG	CTGTGATGCAAGATGCCACT
Upk2	ATCCTGATTCTGCTGGCTGT	GGCACCACAAAGTCTGACTT
Upk3a	CGGCCACTGAGTACAGATTC	GGACGTGATGACAATCATGC
Krt20	TCACAGTGAACACGGAGGAG	ACGAGCCTTGACGTCCTCTA
Krt5	GCCTACATGAACAAGGTGGA	GTTGGCAATGTCCTCGTACT
PPARγ	GGAGTTCATGCTTGTGAAGG	CCTGATGGCATTGTGAGACA
Col IV	TCCAGGCCCCCCTGGAACTGT	GAGGGCCTGGTTGGCCTG
TGFβ1	AGGGCTACCATGCCAACTTC	CCACGTAGTCGATGGGC
S100A4	CAGGCAAAGAGGGTGACAAG	CAATGCAGGACAGGAAGACA
Periostin	GAATGGTGTCATCCACCTGA	GTCCATGCTCAGAGTGTCAT
Desmin	ACCTTCTCTGCTCTCAACTTCC	CGCTGACAACCTCTCCATCC
